# Associations between asthma, overweight and physical activity in children: a cross-sectional study

**DOI:** 10.1186/s12889-016-3600-1

**Published:** 2016-09-01

**Authors:** Maartje Willeboordse, Kim D. G. van de Kant, Charlotte A. van der Velden, Constant P. van Schayck, Edward Dompeling

**Affiliations:** 1 Department of Pediatric Pulmonology, School for Public Health and Primary Care (CAPHRI), Maastricht University Medical Center (MUMC), Maastricht, The Netherlands; 2 Department of Family Medicine, School for Public Health and Primary Care (CAPHRI), Maastricht University, Maastricht, The Netherlands

**Keywords:** Activity, Body mass index, Exercise, Overweight, Pediatrics

## Abstract

**Background:**

Asthma and obesity are highly prevalent in children, and are interrelated resulting in a difficult-to-treat asthma-obesity phenotype. The exact underlying mechanisms of this phenotype remain unclear, but decreased physical activity (PA) could be an important lifestyle factor. We hypothesize that both asthma and overweight/obesity decrease PA levels and interact on PA levels in asthmatic children with overweight/obesity.

**Methods:**

School-aged children (*n* = 122) were divided in 4 groups (healthy control, asthma, overweight/obesity and asthma, and overweight/obesity). Children were asked to perform lung function tests and wear an activity monitor for 7 days. PA was determined by: step count, active time, screen time, time spent in organized sports and active transport forms. We used multiple linear regression techniques to investigate whether asthma, body mass index-standard deviation score (BMI-SDS), or the interaction term asthma x BMI-SDS were associated with PA. Additionally, we tested if asthma features (including lung function and medication) were related to PA levels in asthmatic children.

**Results:**

Asthma, BMI-SDS and the interaction between asthma x BMI-SDS were not related to any of the PA variables (*p* ≥ 0.05). None of the asthma features could predict PA levels (*p* ≥ 0.05). Less than 1 in 5 children reached the recommended daily step count guidelines of 12,000 steps/day.

**Conclusion:**

We found no significant associations between asthma, overweight and PA levels in school-aged children in this study. However, as PA levels were worryingly low, effective PA promotion in school-aged children is necessary.

**Electronic supplementary material:**

The online version of this article (doi:10.1186/s12889-016-3600-1) contains supplementary material, which is available to authorized users.

## Background

**Fig. 1:**
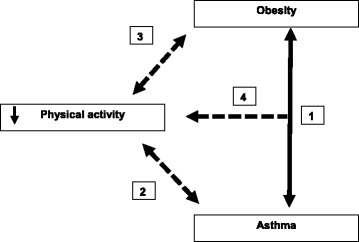
Hypothetical associations between asthma, obesity and decreased physical activity. Pathway 1) Obesity is associated with asthma, and a so-called early-onset asthma-obesity phenotype has been detected [5]. Pathway 2) Asthma is hypothetically associated with decreased physical activity (PA), as it is accompanied with exercise-related limitations. Pathway 3) Obesity is hypothetically associated with decreased PA, as decreased PA is a cause of the imbalance in energy expenditure in obesity. Pathway 4) Obesity and asthma are hypothetically both related to decreased PA levels, therefore it is plausible that an interactive effect of asthma and obesity on decreased PA levels exists

In both adults and children, a positive association between obesity and asthma is demonstrated (Fig. 1, pathway 1) [1–3]. Several studies confirmed that an asthma-obesity phenotype exists, which is dominant among children and middle-aged women [1, 4, 5]. The asthma-obesity phenotype is characterized by high medication use, high admissions rates to the intensive care unit for asthma-related problems and a decreased quality of life [5]. The exact mechanisms that underlie the asthma-obesity phenotype remain unclear. Most studies assume that obesity precedes asthma, although a bidirectional relationship cannot be ruled out [1, 3, 6]. It has been hypothesized that the increased fat mass in obesity causes both mechanical and systemic inflammatory changes which in turn influence breathing mechanisms and airway inflammation [7–9].

Next to the inflammatory and mechanical hypothesis, several lifestyle factors could play an important role in the characterization of the asthma-obesity phenotype. Especially the role of decreased physical activity (PA) deserves further attention, as both asthma and obesity could place children at risk of reduced engagement in physical activity [10]. Approximately 40–90 % of all asthma patients experience exercise induced bronchoconstriction (EIB). Several asthma-related characteristics such as EIB, uncontrolled symptoms and parental beliefs can cause avoidance of PA (Fig. 1, pathway 2) [11, 12]. Obesity is also associated with decreased PA, as decreased PA results in an imbalance in energy expenditure in obesity (Fig. 1, pathway 3).

Several studies investigated PA levels among children with asthma, but conflicting results have been found. Some studies found lower PA levels in children with asthma whilst others reported no difference in PA levels between asthmatic children and healthy peers [10, 11, 13–15]. Fedele et al. studied differences in PA levels between children with obesity, with or without asthma [16]. They found a non-significant trend (p < 0.10) for lower levels of PA in youth in the asthma and obesity group compared to youth in the obesity only group [16].

Limitations of several studies in this field are that factors that influence PA, such as seasonal influences, are not taken into account [10, 17]. Additionally, questionnaires are frequently used to assess PA, which has a low correlation with objective PA measurements [10, 18].

In this study we test the hypothesis that both asthma and overweight/obesity are associated with low PA levels and interact on PA levels in children (Fig. 1, pathway 4). Moreover, we study whether there is a dose-response effect of asthma features (e.g. symptoms and lung function) on decreased PA.

## Methods

### Participants

For this study, 4 groups of children aged 6–12 years were recruited; children with asthma and with overweight/obesity (AO), children with asthma without overweight/obesity (A), non-asthmatic children with overweight/obesity (O) and healthy children without asthma and overweight/obesity (HC). The A, O and HC group were randomly selected from a previous online survey study about respiratory symptoms and anthropometric values. Parents of all children in South Limburg, the Netherlands were invited for this survey in May 2010 (MEC 09-2-088) [19]. The children of the AO group were recruited in 2012 and 2013 from the control group of a randomized controlled trial (RCT) in children with asthma and with overweight/obesity (clinicaltrial.gov NCT00998413) [20]. All children were recruited within six months after the baseline measurement of the RCT.

### Study design

All participants visited the hospital once for anthropometric measurements, completing questionnaires, lung function tests, and a Fraction of exhaled nitric oxide (FeNO) measurement. During this visit all children received an activity monitor with the instruction to wear it for 7 consecutive days, and send it back with a physical activity diary by regular mail.

### Eligibility criteria

A child was considered to be overweight if the BMI- standard deviation score (BMI-SDS) was ≥1.1 [21]. Normal weight was defined as a BMI-SDS ≥ −1.8 and <1.1 according to the reference values of the fourth national Dutch growth study [21]. Furthermore, questionnaires were taken and lung function tests were performed during the hospital visit to diagnose asthma. A child was considered asthmatic if the participant fulfilled at least 2 of the following 3 criteria:

1. An asthma diagnosis given by a general practitioner or pediatrician;
2. Current asthma symptoms (wheezing, or dry cough at night during the previous year) or use of asthma medication during the previous year (short acting beta-2agonists (SABA), long acting beta-2agonists (LABA) and/or inhaled corticosteroids (ICS))
3. Reversibility of airway obstruction on a bronchodilator. This was defined as ≥9 % improvement in forced expiratory volume in 1 s (FEV_1_) of the predicted value after 400 μg salbutamol (Teva Pharma, Leiden, the Netherlands).

Children were considered non-asthmatic if they were never diagnosed with asthma by a doctor and had not used asthma medication in the previous year. Children were excluded from the study in case of a congenital malformation of the airways or other chronic lung diseases, syndromes, or physical limitations that make intense PA impossible.

### Anthropometric measurements

Children’s body weight and height were assessed twice to the nearest 0.1 kg and 0.5 cm, while wearing underwear, by using a medical calibrated weight scale and stadiometer (Model 877 and 213, Seca, Hamburg, Germany). The BMI-SDS was calculated with reference values of the fourth national Dutch growth study as described above [21].

### Lung function, airway inflammation and respiratory complaints

Maximal expiratory flow volume curves were obtained with the ZAN100 (ZAN Messgerate, Oberthulba, Germany), according to international standardized guidelines [22, 23]. LABA was withheld 48 h and SABA 8 h before the measurement. FeNO was obtained with the online NIOX analyzer (Aerocrine, Solna, Sweden) according to international guidelines [24].

Information about respiratory complaints was collected by using a standardized questionnaire on respiratory complaints (International study of asthma and allergies in childhood questionnaire) [25]. Asthma control was measured by the childhood-asthma control test (c-ACT), with a cut-off value of ≤19 for uncontrolled asthma [26]. In addition, questionnaires were used to determine respiratory complaints and the use of asthma medication in the previous 2 months [25]. Dose equivalents were calculated according to standard dosages of SABA and ICS [27, 28].

### Physical activity measurements

Step count was measured by a triaxial accelerometer (Yamax EX510 Power Walker, Yamax, Tokyo, Japan) within 2 weeks after the hospital visit. Outcomes of activity monitors produced by Yamax were demonstrated to be valid and reproducible [29]. Children were instructed by a trained researcher to wear the activity monitor for 7 consecutive days during waking hours. The activity monitor was worn in children’s pocket, according to the instructions of the Yamax manufacturer. Data were considered missing when participants wore the activity monitor less than 3 weekdays and 1 weekend day. The output of the activity monitors was analyzed in step count/day and in minutes active/day. We calculated if children met the daily PA recommendations of at least 12,000 steps/day [30], and whether children were less, equally or more active compared to a reference population of Canadian children [31]. The time spent in PA was calculated by adding active time measured by the activity monitor, cycling and swimming time. The latter two were derived from a 7-day activity diary which the children were instructed to fill in with their parent(s) while wearing the activity monitor.

Screen time, time spent in organized sport activities (without school-regulated sport activities) and active transport forms to school were derived from a Dutch questionnaire that parents fulfilled during the hospital visit [32]. Furthermore, the attitudes towards PA and exercise-related limitations were assessed with a parental questionnaire and an interviewer administered questionnaire for the child.

### Statistical analysis

SPSS (version 20.0) was used for analysis. Baseline characteristics between the groups were compared by means of ANOVA, relevant post-hoc differences between groups were listed in the results section. There were no missing values, except for several PA values. Missing PA values (*n* = 29, 5.9 % of the selected PA variables) were imputed by using multiple imputation techniques with 5 imputations (see Additional file 1: Table S1). Data was analyzed with the assumption that data was missing at random, as the majority of the baseline values did not statistically differ between participants with and without missing data. The only variables that differed between the two groups were season of the measurement and control of asthma (see Additional file 1: Table S2).

Variables included in the imputation model were: step count, time spent in PA, screen time, time spent in organized sport activities and active transport forms. Reasons for missing data included lost or broken activity monitors, illness, incomplete data and not returning the activity monitor despite multiple reminders. Imputated data is shown in the Result section, unless otherwise stated.

Several multiple linear regressions were used to investigate the influence of asthma, BMI-SDS and the interaction term asthma x BMI-SDS on the various PA variables (step count, time spent in PA, screen time, time spent in organized sport activities and active transport forms). The variables age, sex and season of measurement (as calculated by the metrological calendar) were added to the model by the enter method as they are believed to be correlated with individual PA levels in children [17, 33].

To answer the second research question, we performed multiple linear regression analyses (enter method) in all children with asthma. We tested whether c-ACT score, FEV_1_%predicted, FeNO, airway reversibility (in percentage), dose equivalent of SABA or parental and children’s perception of asthma-related exercise limitations could predict the various PA variables. A significance level of less than 0.05 was used for all analyses.

### Sample size calculation

A sample size estimation for multiple group comparisons using analysis of variance was performed. As we expected the smallest differences in PA between the O and HC group and A and HC group, we based our calculation on a previous study that found a difference between O and HC group of 3669 steps (SD = 5576) [34]. In total, 116 children (29 per group) are sufficient to detect a significant difference in average daily step count with a power of 80 %, a two-sided alpha of 0.05 and a drop-out rate of 10 %.

## Results

### Baseline characteristics

PA measurements were conducted during regular school weeks between May 2012 and February 2013. Baseline characteristics are presented in Table 1. All children were Caucasian. Forty percent of the asthmatic children showed airway reversibility and the majority of the asthmatic children had well controlled asthma and used maintenance asthma medication. Groups differed slightly in age, with significantly younger children in the A and HC groups and older children in the AO and O groups. Relatively more boys were included in the A and AO groups than in the HC and O group. Children in the AO group had a significantly wider waist circumference than the children in the O group, but did not differ in other anthropometric values compared to the O group. Asthma-related variables were not different between A and AO groups, except for a significantly higher number of children in the AO group which used more than 1 dose equivalent of ICS.

**Table 1:** Baseline characteristics

	Total group(*n* = 122)	HC(*n* = 33)	A(*n* = 29)	O(*n* = 30)	AO(*n* = 30)
Baseline variables					
Age in years, median (IQR)*	10.8 (2.2)	10.3 (2.1)	10.1 (2.5)	11.2 (1.9)	11.9 (2.1)
Sex boys/girls, %*	52/48	36/64	69/31	40/60	63/37
Measurement during summer season, %	22	27	24	10	27
PA variables					
Step count, mean (SD)	9535 (3300)	9304 (3063)	11,150 (4186)	8383 (2906)	9302 (2287)
Meeting at least 12,000 steps/day, %	19	21	36	11	10
Equally, or more active as reference population [30], %	36	28	48	25	42
Anthropometric variables					
BMI in kg/m^2^, median (IQR)*	18.8 (7.0)	16.5 (3.0)	16.1 (1.5)	22.9 (1.8)	23.6 (5.5)
BMI-SDS, median (IQR)*	0.93 (2.28)	−0.11 (1.31)	−0.21 (0.90)	1.94 (0.52)	2.20 (0.76)
Hip circumference in cm, mean (SD)*	78.1 (11.7)	70.0 (6.7)	68.3 (6.4)	86.5 (7.4)	88.6 (8.3)
Waist circumference in cm, median (IQR)*	69.5 (19.9)	59.5 (12.4)	60.8 (8.9)	78.5 (9.0)	85 (20.4)
Asthma-related variables					
FEV_1_ %predicted, mean (SD)*	96 (16)	103 (14)	93 (10)	102 (12)	85 (20)
Airway reversibility, %*	22	0	31	10	50
Uncontrolled asthma, %	10	n.a.	8	n.a.	14
Asthma medication use, %*	33	0	69	0	67
SABA use, % (% of those children using more than 1 dose equivalent)*	25 (42)	0 (0)	59 (35)	0 (0)	47 (50)
ICS use, % (% of those children using more than 1 dose equivalent)*	23 (32)	0 (0)	52 (21)	0 (0)	43 (46)
FeNO in ppb, median (IQR)*	13.0 (12.6)	12.9 (7.4)	19.0 (27.4)	10.6 (5.0)	20.9 (18.5)

*Abbreviations*: *BMI* body mass index, *BMI-SDS* body mass index-standard deviation score, *FeNO* fraction of exhaled nitric oxide, *FEV*_*1*_ forced expiratory volume in 1 s, *ICS* inhaled corticosteroids, *IQR* interquartile range, *n.a.* not applicable, *SABA* short acting beta2-agonists, *SD* standard deviation

*Significant differences between groups with *p* < 0.05, measured by ANOVA

### Physical activity levels

**Fig. 2:**
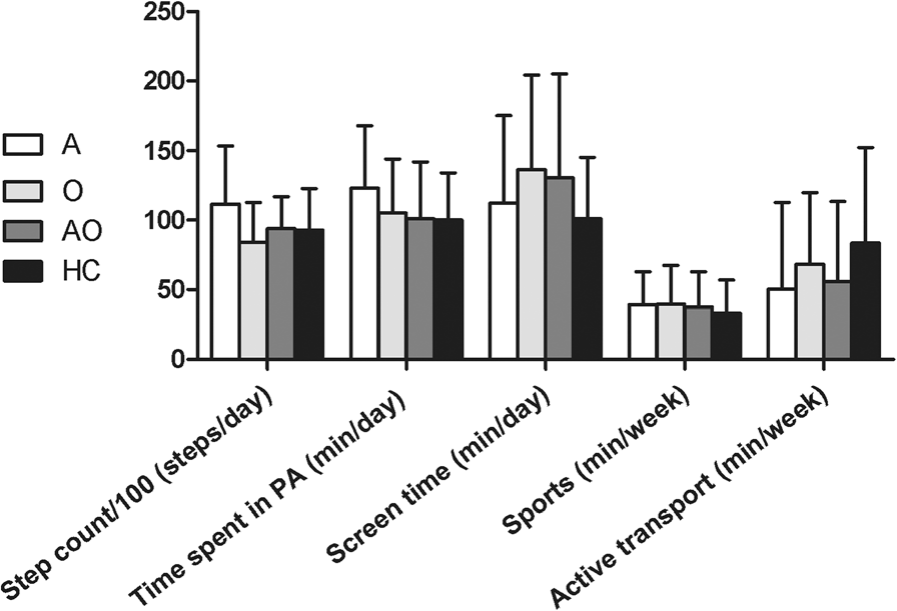
Physical activity levels per group. There were no significant differences between groups in each of the categories, measured by ANOVA (*p* ≥ 0.05). Abbreviations: A: asthma, O: overweight/obesity, AO: asthma and overweight/obesity, HC: healthy control, PA: physical activity

Less than 1 in 5 children met the current step count guidelines for school-aged children of 12,000 steps/day (Table 1), and children spent on average less than 60 min in organized sport activities outside school (Fig. 2). Compared to a Canadian reference population, the majority of the children was less active (Table 1). Children measured in the summer, made on average 2307 more steps/day (*p* < 0.01), and spent on average 41 more minutes in PA/day, than children measured during the autumn (Table 2). Boys made on average 2489 more daily steps than girls (*p* < 0.05) and children spent on average 10.7 min more in weekly active transport forms to school for every year they were older (*p* < 0.05) (Table 2).

**Table 2:** Standardized B values for the prediction models of several PA variables

Dependent variable	BMI-SDS	Asthma	Asthma x BMI-SDS	Age	Sex^a^	Autumn season^b^	Step count
Step count	−.12	.08	−.03	−.18	−.30*	−.36*	n.a.
Time spent in PA	.04	.02	−.02	.14	−.03	−.28*	.54*
Screen time	.17	<−.01	−.10	.11	.02	−.11	−.06
Time spent in organized sports	.06	−.06	−.07	.03	−.18	.06	.31*
Time spent in active transport to school	−.09	−.19	.04	.23^*^	−.09	−.11	−.10

*Abbreviations*: *BMI* body mass index, *BMI-SDS* body mass index – standard deviation score, *n.a.* not applicable, *PA* physical activity

*Significant correlation (*p* < 0.05) with the dependent variable

^a^Boys are coded as 0, girls as 1, ^b^As opposed to the summer season

### Physical activity, asthma and BMI-SDS

Neither asthma nor BMI-SDS was correlated to any of the PA variables (Table 2, Fig. 2). Also the interaction term asthma by BMI-SDS could not explain variability in PA levels. When compared to the model without imputations, interpretation of the tests remained unchanged, except for the variable age which was a significant predictor for step count in the model without imputations, but not in the model with imputations (see Additional file 1: Table S3).

### Physical activity and asthma features

In the group of asthmatic children, we assessed whether asthma features could explain variability in PA levels. The overall model fitting was low as R squares ranged from 0.05 to 0.20. Asthma control, FEV_1_% predicted value, airway reversibility, SABA dose equivalent, FeNO, and perception of asthma-related exercise limitations by both child and parents could not predict any of the PA variables (*p* ≥ 0.05) (Table 3). When compared to the model without imputations, interpretation of the tests remained unchanged (see Additional file 1: Table S4).

**Table 3:** Standardized B values for the relation between asthma related parameters and PA levels

Dependent variable	c-ACT	FEV_1_%	Airway reversibility	SABA dose equivalent	Parental perception	Child’s perception	FeNO
Step count	−.07	.03	−.18	−.08	.01	−.02	.16
Time spent in PA	−.01	−.18	−.02	<.01	−.14	−.11	.16
Screen time	−.09	−.25	.06	−.07	.11	.16	.06
Time spent in organized sports	−.06	−.14	−.15	−.09	.07	−.19	−.06
Time spent in active transport to school	−.13	−.26	.09	.03	−.29	−.06	.14

*Abbreviations*: *c-ACT* childhood-asthma control test, *FeNO* fraction of exhaled nitric oxide, *FEV*_*1*_ *%* forced expiratory volume in 1 s in percentage of predicted, *PA* physical activity, *SABA* short acting beta2-agonists

## Discussion

The main finding of our study is that the level of physical activity in all children was alarmingly low, but there was no indication for an interactive effect of overweight/obesity and an asthma diagnosis on physical activity levels. Moreover, asthma features and attitudes towards asthma-related exercise limitations by both parents and children were not related to PA levels.

We hypothesized that children with asthma would have decreased PA levels, as asthma is associated with exercise-related limitations (Fig. 1). In addition, we hypothesized that a dose-response effect of several asthma features on decreased PA levels would be visible in asthmatic children. Although we measured PA by several outcome measures, neither asthma diagnosis nor asthma features were associated with any of the PA outcome measures.

One explanation for the lack of effect of either asthma, asthma features or BMI-SDS, is that PA levels were considerably low in all groups, resulting in hardly any ‘room for worsening’. Less than 20 % of all children met the minimum recommended daily step count guidelines. Compared to a reference population of Canadian school-aged children, the majority of our study population had a lower step count [31]. Not only step count, but also other PA variables were alarmingly low, as children spent more minutes in sedentary position behind screens than being physically active (Fig. 2). This implies that despite several attempts of Western politics to increase children’s PA, levels still remain too low, which could lead to considerable health threats.

Although it is plausible that we did not found deteriorations in PA levels in asthma or overweight/obesity as PA levels are already low, it is conceivable that we should refute our initial hypothesis (Fig. 1). As we found that not only an asthma diagnosis, but also asthma features were not associated with PA, it seems unlikely that asthma is associated with decreased PA levels. Several other studies investigated the possible association of asthma with PA levels in children, but conflicting results have been found. Tsai et al. reported 12 cross-sectional studies, their study included, that measured PA levels in children with and without asthma [10]. Six studies found decreased PA levels, 5 studies found equal PA levels and 1 study found increased PA levels in children with asthma or history of wheezing compared to healthy controls [10]. Neither differences in methodology (e.g. different PA outcome measures), population characteristics, country or comorbidities could explain the inconsistencies in those studies. The findings of our and other studies cannot explain why childhood asthma is in some cases, but not all, associated with decreased PA.

The association between obesity and reduced PA has been found in several [18, 35], but not all studies [36]. Possibly, obesity is not always related to PA levels, as in children, obesity might be primarily caused by an increase in energy intake, and not by a decrease in energy expenditure [37]. A meta-analysis of Wilks et al. confirmed that individual differences in PA are often not associated with obesity in childhood, as a healthy diet plays a more important role in childhood obesity than PA levels [36]. Besides, it is possible that our results have been influenced by Dutch asthma guidelines, that promote overweight prevention and PA stimulation actively in children with asthma [28].

Although PA does not seem to be correlated to current asthma, decreased PA may play a role in the development of asthma. Sheriff et al. and Rasmussen et al. demonstrated that low PA levels and frequent television viewing were associated with the development of asthma from pre-school and school-age into adolescence, independent of BMI status [38, 39]. In addition, a meta-analysis in adults showed that subjects with high PA levels have a lower risk to develop asthma (OR: 0.87 (95 % CI 0.77–0.99)) [40].

A strength of this study is the inclusion of 4 groups of children, which made it possible to investigate the interaction effect of asthma and overweight/obesity. Secondly, we measured both asthma and PA objectively in this study, in contrast with most other studies which frequently use self-reported outcome measures [10]. However, some potential limitations should be mentioned. Firstly, children with asthma and with overweight were recruited from the control group of a weight-reduction intervention study [20]. Possibly, these children had increased PA levels as they were initially interested in a weight-reduction intervention. Secondly, we used activity monitors which could not distinguish moderate from intense PA. This is a potential study limitation as it is conceivable that exercise-related limitations of asthma and overweight/obesity are most pronounced during high intensity activities. However, as organized sport participation measured by a questionnaire was not associated with either asthma or BMI-SDS, this hypothesis can most likely be refuted. Thirdly, the age range of our study population could have influenced variability in PA levels, as individual differences in PA are quite small during childhood due to school-regulated activity patterns. Finally, a lack of power could also have hampered our results. Due to the low average step count of our population, it was relatively difficult to detect a statistical difference in step count between the groups.

We could not confirm our hypothesis that both asthma and BMI-SDS are associated with decreased PA. The exact role of PA in the asthma-obesity mechanism remains largely unclear. However, as the asthma-obesity phenotype is a difficult-to-threat phenotype with mostly non-modifiable risk factors, we want to emphasize the need to further investigate the role of potentially modifiable risk factors such as PA and diet in large cohort studies. Moreover, it is highly relevant to assess whether increasing PA could be a secondary preventive tool in children with asthma and overweight/obesity [20]. Although the effects of exercise training on lung function in asthmatic children are negligible, there is evidence of positive effects on cardiorespiratory fitness, EIB and potentially on quality of life and asthma control [41]. Besides, several studies in adults showed that weight reduction resulted in improved asthma control, lung function, and less medication use [42].

## Conclusions

We could not detect different PA levels in school-aged children with asthma and overweight/obesity compared to peers without asthma and/or overweight/obesity. Yet, it should be mentioned that PA levels were alarmingly low in the entire study population. As decreased PA levels are related to higher health risks, we emphasize the importance of improving PA levels in school-aged children. Future studies should focus on effective interventions to increase PA in school-aged children. Besides, it should be studied whether increasing PA could be an effective secondary preventive tool for children with asthma and with overweight/obesity.

## Additional file

Additional file 1: Table S1. Amount of multiple imputations for all PA variables. **Table S2** Baseline differences between individuals with and without imputations. **Table S3** Standardized B values for the prediction models without multiple imputation techniques. **Table S4**: Standardized B values for the relation between various asthma related parameters with PA levels without multiple imputation techniques. (DOCX 22 kb)

## Abbreviations

A: Group of children with asthma
AO: Group of children with asthma and overweight/obesity
ATS/ERS: American thoracic society / European respiratory society
BMI: Body mass index
BMI-SDS: Body mass index – standard deviation score
c-ACT: Childhood-asthma control test
EIB: Exercise-induced bronchoconstriction
FeNO: Fraction of exhaled nitric oxide
FEV1%: Forced expiratory volume in 1 s, in % of predicted
HC: Group of healthy controls, e.g. children without asthma and overweight/obesity
ICS: Inhaled corticosteroids
IQR: Inter quartile range
LABA: Long acting beta2-agonists
O: Group of children with overweight/obesity
PA: Physical activity
SABA: Short acting beta2-agonists
SD: Standard deviation
SE: Standard Error
