# Supplementary material for: Associations between asthma, overweight and physical activity in children: a cross-sectional study
Source: BMC Public Health. 2016 Sep 1;16(1):919. doi: 10.1186/s12889-016-3600-1 (PMC5009538; doi:10.1186/s12889-016-3600-1)
Supplement: Additional file 1: Table S1. — Amount of multiple imputations for all PA variables. Table S2 Baseline differences between individuals with and without imputations. Table S3 Standardized B values for the prediction models without multiple imputation techniques. Table S4: Standardized B values for the relation between various asthma related parameters with PA levels without multiple imputation techniques. (DOCX 22 kb) [file 12889_2016_3600_MOESM1_ESM.docx]

**Additional file 1**

**Table S1: Amount of multiple imputations for all PA variables**

| **PA variables** | **Amount of missing variables, (% of total)** |
| --- | --- |
| Step count | 10 (8.2) |
| Time spent in PA | 12 (9.8) |
| Screen time | 4 (3.3) |
| Time spent in organized sport activities | 3 (2.5) |

Abbreviations: PA: physical activity.

**Table S2: Baseline differences between individuals with and without imputations**

|  | **Original dataset**  **(n=122)** | **Participants without imputated values (n=101)** | **Participants with imputated values**  **(n=21)** |
| --- | --- | --- | --- |
| *Baseline variables* |  | | |
| Age in years, median (IQR) | 10.8 (2.2) | 10.8 (2.3) | 11.6 (2.1) |
| Sex male/female, % | 52 / 48 | 54 / 45 | 33 / 67 |
| Measurement during summer season, %* | 22 | 21 | 29 |
| *Anthropometric variables* |  | | |
| BMI in kg/m^2^, median (IQR)_­_ | 18.8 (7.0) | 18.6 (6.9) | 22.4 (7.0) |
| BMI-SDS, median (IQR) | 0.93 (2.28) | 0.82 (2.33) | 1.64 (1.87) |
| Hip circumference in cm, mean (SD) | 78.1 (11.7) | 77.5 (11.4) | 73.0 (20.5) |
| Waist circumference in cm, median (IQR) | 69.5 (19.9) | 69.2 (19.5) | 80.9 (13.0) |
| *Asthma-related variables* |  | | |
| FEV_1_ %predicted (SD) | 96 (16) | 96 (15) | 94 (20) |
| Airway reversibility, % | 22 | 22 | 24 |
| Uncontrolled asthma, %* | 10 | 6 | 38 |
| Asthma medication use, % | 33 | 35 | 24 |
| SABA use, % | 25 | 26 | 24 |
| ICS use, % | 23 | 26 | 10 |
| FeNO in ppb, median (IQR) | 13.0 (12.6) | 14.0 (14.6) | 11.0 (6.8) |

* Significant difference between groups (p<0.05). Abbreviations: BMI: body mass index, BMI-SDS: body mass index-standard deviation score, FeNO: fraction of exhaled nitric oxide, FEV_1_: forced expiratory volume in 1 second, ICS: inhaled corticosteroids, IQR: inter quartile range, SABA: short acting beta2-agonists, SD: standard deviation.

**Table S3: Standardized B values for the prediction models without multiple imputation techniques.**

| **Dependent variable** | **BMI-SDS** | **Asthma** | **Asthma x BMI-SDS** | **Age** | **Sex^#^** | **Autumn season^^^** | **Step count** |
| --- | --- | --- | --- | --- | --- | --- | --- |
| Step count | -.14 | .07 | -<.01 | -.22* | -.33* | -.39* | n.a. |
| Time spent in PA | .02 | <.01 | <-.01 | .17 | -.01 | -.27* | .58* |
| Screen time | .22 | .03 | -.14 | .12 | .04 | -.09 | -.03 |
| Time spent in organized sports | .02 | -.03 | -.04 | <-.01 | -.18 | .03 | .29* |

* Significant correlation (p<0.05) with the dependent variable, # Boys are coded as 0, girls as 1,

^^^ As opposed to the summer season. Abbreviations: BMI-SDS: body mass index – standard deviation score, n.a.: not applicable, PA: physical activity.

**Table S4: Standardized B values for the relation between various asthma related parameters with PA levels without multiple imputation techniques.**

| **Dependent variable** | **c-ACT** | **FEV_1_%** | **Airway reversibility** | **SABA dose equivalent** | **Parental percep-tion** | **Child’s percep-tion** | **FeNO** |
| --- | --- | --- | --- | --- | --- | --- | --- |
| Step count | -.06 | -.02 | -.14 | -.07 | -.01 | <-.01 | .18 |
| Time spent in PA | -.06 | -.20 | -.03 | <.01 | -.18 | -.10 | .16 |
| Screen time | -.09 | -.21 | <.01 | -.10 | .12 | .11 | .03 |
| Time spent in organized sports | -.04 | -.12 | -.14 | -.08 | .08 | -.17 | -.02 |

* Significant correlation (p<0.05) with the dependent variable, Abbreviations: c-ACT: childhood asthma control test, FeNO: fraction of exhaled nitric oxide, FEV_1_%: forced expiratory volume in 1 second in % of predicted, PA: physical activity, SABA: short acting beta2-agonists.
